# Sources of Muscular Power

**Published:** 1878-09

**Authors:** 


*On the Source of Muscular Power; Arguments and Conclusions drawn from Observations upon the Human Subject under Conditions of Rest and of Muscular Exercise.* By AUSTIN FLINT, JR., M. D. New York: D. Appleton & Co. 1878.

This Essay was first printed in the Journal of Anatomy and Physiology in Oct. 1877. In its present form several errors which unfortunately were present in its first publication have been corrected and some additional matter incorporated with the essay. Dr. Flint does not hold with the theories now taught by many that organic life and all its processes can be simply and sufficiently explained by the laws, as far as we understand them, which govern inorganic life.

The question which he set about to solve is one which, though apparently simple,, is so surrounded and involved by complex physiological phenomena that the problem becomes one of no small magnitude.

The question directly involved is: Does food by its transformation become directly concerned in the production of muscular power or does "muscular effort involve changes in the muscular substance itself, this substance being destroyed as muscular tissue, discharged from the body in the form of excrementitious matter, and the waste being repaired by food." In the solution of this question Prof Flint gives in full the details of experiments and observations made upon the pedestrian Weston. We have not space to follow these interesting experiments but will say that they were carefully made and the results of observation conscientiously recorded.

Dr. Pavy in some experiments made under similar circumstances with Weston has reached results which differ materially from those here recorded, but Dr. Flint shows in several instances palpable sources of error in Pavy's investigations. The work is one which will be read with interest and coming from the source it does, cannot but have a large bearing upon the question. We have only room to give the authors conclusions, which are summarized in the following paragraph :

Finally, experiments upon the human subject show that the direct source of muscular power is to be looked for in the muscular system itself. The exercise of muscular power immediately involves the destruction of a certain amount of muscular tissue of which the nitrogen excreted is a measure. Indirectly nitrogenized food is a source of power, as, by its assimilation by the muscular tissue, it repairs the waste and develops the capacity for work; but food is not converted into force in the living body, nor is it a source of muscular power except as it maintains the muscular system in a proper condition for work.

We advise those of our readers interested in the subject to read the work. They will find in a small space a large amount of valuable information, carefully drawn from well conducted observation.

